# Detection of inflammation by whole-body MRI in young people with juvenile idiopathic arthritis

**DOI:** 10.1093/rheumatology/keae039

**Published:** 2024-01-20

**Authors:** Varvara Choida, Timothy J P Bray, Niels van Vucht, Maaz Ali Abbasi, Alan Bainbridge, Thomas Parry, Debajit Sen, Sue Mallett, Coziana Ciurtin, Margaret A Hall-Craggs

**Affiliations:** Centre for Medical Imaging, University College London, University College London, London, UK; Centre for Adolescent Rheumatology, Division of Medicine, University College London, London, UK; Department of Rheumatology, University College London Hospitals NHS Foundation Trust, London, London, UK; Centre for Medical Imaging, University College London, University College London, London, UK; Department of Imaging, University College London Hospitals NHS Foundation Trust, London, UK; Department of Imaging, University College London Hospitals NHS Foundation Trust, London, UK; Department of Imaging, University College London Hospitals NHS Foundation Trust, London, UK; Centre for Medical Imaging, University College London, University College London, London, UK; Department of Medical Physics, University College Hospitals Trust, London, UK; Centre for Medical Imaging, University College London, University College London, London, UK; Centre for Adolescent Rheumatology, Division of Medicine, University College London, London, UK; Department of Rheumatology, University College London Hospitals NHS Foundation Trust, London, London, UK; Centre for Medical Imaging, University College London, University College London, London, UK; Centre for Adolescent Rheumatology, Division of Medicine, University College London, London, UK; Department of Rheumatology, University College London Hospitals NHS Foundation Trust, London, London, UK; Centre for Medical Imaging, University College London, University College London, London, UK; Department of Imaging, University College London Hospitals NHS Foundation Trust, London, UK

**Keywords:** whole-body, MRI, JIA, synovitis, outcomes, disease activity, MMP-3, VEGF

## Abstract

**Objectives:**

To assess the frequency of joint inflammation detected by whole-body MRI (WBMRI) in young people (YP) with JIA and controls, and to determine the relationship between WBMRI-detected inflammation and clinical findings.

**Methods:**

YP aged 14–24 years, with JIA (patients) or arthralgia without JIA (controls), recruited from one centre, underwent a WBMRI scan after formal clinical assessment. Consensus between at least two of the three independent radiologists was required to define inflammation and damage on WBMRI, according to predefined criteria. YP with JIA were deemed clinically active as per accepted definitions. The proportions of YP with positive WBMRI scans for joint inflammation (one or more inflamed joint) as well as serum biomarkers were compared between active *vs* inactive JIA patients and controls.

**Results:**

Forty-seven YP with JIA (25 active and 22 inactive patients) and 13 controls were included. WBMRI detected joint inflammation in 60% (28/47) of patients with JIA *vs* 15% (2/13) of controls (difference: 44%, 95% CI 20%, 68%). More active than inactive JIA patients had WBMRI-detected inflammation [76% (19/25) *vs* 41% (9/22), difference: 35% (95% CI 9%, 62%)], and this was associated with a specific biomarker signature. WBMRI identified inflammation in one or more clinically inactive joint in 23/47 (49%) patients (14/25 active *vs* 9/22 inactive JIA patients).

**Conclusions:**

WBMRI’s validity in joint assessment was demonstrated by the higher frequency of inflammation in JIA patients *vs* controls, and in active *vs* inactive JIA patients. WBMRI found unsuspected joint inflammation in 49% YP with JIA, which needs further investigation of potential clinical implications.

Rheumatology key messages

- The detection of joint inflammation in more JIA patients than controls supports the validity of whole-body MRI (WBMRI).
- WBMRI detected joint inflammation in clinically quiescent joints in 49% of young people with JIA.
- Further research is required to assess the relevance of clinically concordant versus non-concordant WBMRI-detected inflammation.

## Introduction

JIA is an umbrella term that includes multiple subtypes of inflammatory arthritis developing in childhood [1, 2]. The cardinal manifestation is synovitis, but there is great heterogeneity between the disease subtypes [3] in the number and type of joints affected and the presence of additional features, including enthesitis [4], sacroiliitis and spinal inflammation.

In clinical practice, the disease activity of JIA is measured with a combination of patient- and physician-reported outcomes, clinical examination and laboratory markers, which include the patient/parent global assessment of well-being visual analogue score (PtGA), Childhood Health Assessment Questionnaire (CHAQ), physician’s global assessment of disease activity visual analogue score (PhGA), active joint count (AJC), limited joint count (LJC), ESR and/or CRP, and the composite outcome Juvenile Arthritis Disease Activity Score (JADAS) [5].

In addition to the clinical assessments, there is a growing role for imaging in the diagnosis and monitoring of JIA across age groups [6]; for example, MRI is essential for the diagnosis of sacroiliitis [7]. Moreover, many studies have shown the ability of MRI to detect joint inflammation in joints that were clinically inactive (subclinical), including the knee [8], hip [9], wrist [10] and temporomandibular joints (TMJs) [11]. Therefore, MRI could be a valuable tool for monitoring and optimizing disease control and preventing structural damage. The use of whole-body MRI (WBMRI) enables the assessment of multiple joints, the entheses and the spine for inflammation and structural damage in patients with inflammatory arthritis [12]. It could provide a comprehensive assessment of inflammation, suitable for all JIA subtypes, in a single examination. Retrospective studies have shown that WBMRI detects subclinical joint inflammation in children with JIA [13, 14], suggesting that it could be used for personalized therapy in JIA. However, these studies have largely focused on highly selected JIA populations, and the extent to which WBMRI-detected joint inflammation signifies ‘real’ disease *vs* false positives (or synovitis due to other factors) is currently unclear.

To address this evidence gap, we compared the prevalence of MRI-detected inflammation between patients with JIA and ‘control’ patients with musculoskeletal symptoms but without JIA. We hypothesized that WBMRI detects joint inflammation more commonly in patients with JIA than controls.

Our objectives were: (i) to measure and compare the frequency of joint inflammation detected on WBMRI in young people (YP) with JIA and controls, and (ii) to assess the relation between clinical, laboratory and imaging markers of disease activity in JIA.

## Methods

### Study design and participants

This cross-sectional study was approved by the London Queen Square Research Ethics Committee (15/LO/1475). All participants gave written informed consent. The study complied with the ethical principles of the Declaration of Helsinki. Participants were recruited prospectively from the adolescent and young adult rheumatology department of University College London Hospital. The eligibility criteria for the patient group included a diagnosis of JIA (any subtype) and age between 12 and 24 years. There were no exclusion criteria regarding their treatment or disease activity. The inclusion criteria for the control group were musculoskeletal pain in the absence of a diagnosis of JIA according to the opinion of the rheumatologist, and age between 12 and 24 years. The WBMRI was not performed for clinical reasons. Patients with contraindications for MRI or gadolinium were excluded.

### Clinical assessment

All participants were assessed clinically on the day of their WBMRI examination, by the same experienced examiner. The clinical disease activity measures collected included the AJC, LJC, PtGA, PhGA and CHAQ. Active sacroiliitis was determined according to the definition used in the juvenile SpA disease activity as the presence of at least two of the three criteria: inflammatory back pain, sacroiliac joint (SIJ) tenderness and positive Patrick’s test [15]. Based on the clinical assessment (CA), the patients with JIA were divided into two groups; the active group if the ACJ ≥ 1 and/or there was clinical sacroiliitis, and the inactive group if there was none of the above. Patients in the inactive JIA group were assessed against the Wallace criteria for clinically inactive disease [16]. Entheseal tenderness was assessed in 33 sites (Supplementary Data S1, available at *Rheumatology* online). In addition, participants reported their symptomatic joints using a homunculus.

### Imaging acquisition

All participants underwent a WBMRI scan on a 3T MRI scanner (Ingenia, Philips Healthcare, Best, Netherlands). The WBMRI protocol included coronal gradient echo Dixon images acquired after the administration of 10 ml gadoteric acid meglumine. The total scan duration, from the positioning of the participant to the acquisition of the post-contrast images, was ∼30 min. The scanning parameters of the protocol are described in Supplementary Data S2, available at *Rheumatology* online.

### Imaging assessment

Three experienced musculoskeletal radiologists (M.A.H.-C., M.A.A., N.V.) reviewed all participants’ images independently, blinded to clinical information. The post-contrast Dixon images were assessed for joint inflammation, structural damage and enthesitis. Eighty-one joints (71 joints included in JADAS-71 [17], 8 DIP joints of feet and 2 SIJs) were assessed for joint inflammation and structural damage.

Each reader assessed the SIJ and cervical spine (C-spine) for joint inflammation dichotomously (present/absent). Peripheral joints were assessed for synovitis (grade 0–2); only grade 2 synovitis was considered positive for peripheral joint inflammation. Post-contrast above-normal intensity contrast enhancement (grade 1 synovitis) is not a reliable sign of joint inflammation [18]. Grade 2 synovitis was defined as the presence of above-normal intensity post-contrast synovial enhancement, in addition to one of the following characteristics: synovial hypertrophy, subarticular bone marrow oedema, joint effusion or periarticular soft tissue oedema. Details of definitions for joint inflammation, structural damage and enthesitis are provided in Supplementary Data S2, available at *Rheumatology* online.

The imaging assessments by the three readers were combined in one dataset where joint inflammation, enthesitis and structural damage at any joint/entheseal site were recorded as positive if detected by two or more of the three independent WBMRI readers.

### Blood markers of disease activity

Blood samples were collected on the day of the WBMRI scan for the measurement of CRP, ESR and 12 other biomarkers. Luminex assay was used for the measurement of serum MMP-3, S100 calcium-binding protein A8, IL-6, IL-17, macrophage migratory inhibitory factor, IL-23, IL-33, GM-CSF, TNF-α, CD40 ligand, VEGF and IFN-γ (details on sample analysis are available in Supplementary Data S3, available at *Rheumatology* online).

### Outcomes

We measured the proportion of YP with joint inflammation (in one or more joint), structural damage (in one or more joint) and enthesitis (in one or more entheseal site) on WBMRI in the JIA and control groups.

We also measured the proportion of patients with joint inflammation in specific joints/joint groups. Two joint groups, the hand and the forefoot, were created to encompass the 28 small joints of the hand and 28 small joints of the forefoot, respectively.

We compared the CA with the WBMRI assessment for joint inflammation in 81 joints per patient. There were four possible outcomes from this comparison for each joint (+ = positive result, – = negative result): WBMRI+ CA+, WBMRI+ CA–, WBMRI– CA+ and WBMRI– CA–. We measured the frequency of each outcome for each joint. We refer to the WBMRI+ CA– outcome as subclinical inflammation.

We measured the joint counts with WBMRI-detected inflammation and subclinical inflammation in each patient group.

### Statistical analysis

Joints that were not adequately imaged according to the radiologists were excluded from the analysis (Supplementary Data S2, available at *Rheumatology* online). The proportions of patients with joint inflammation, structural damage and enthesitis were compared between the JIA and control group using the unpaired proportions test, respectively, using 95% CI to determine significance. The Mann–Whitney *U* test was used to compare continuous variables. The Spearman’s rank correlation coefficient was used to assess the relation between continuous variables. *P*-values <0.05 were defined as statistically significant.

This is an exploratory study, with no previous literature data available to enable us to determine the effect size of joint inflammation in JIA *vs* controls in this age group, therefore we estimated the sample size as 60 participants based on practical considerations around recruitment.

## Results

### Participants’ characteristics and CA

Forty-seven YP with JIA and 13 controls were recruited between September 2019 and March 2021. The characteristics of both groups are described in Table 1.

**Table 1.** Demographics and measures of JIA disease activity in participants with JIA and controls

Variable	Controls (*n* = 13)	All JIA (*n* = 47)
Age, years	16 (16–17)	18 (16–20)
Male, *n* (%)	2 (15)	18 (38)
Duration of symptoms, years	4.0 (2.0–10.0)	10.0 (6.0–14.5)
CHAQ (0–3)	1.00 (0.63–2.00)	0.50 (0.00–1.38), *n* = 46
Entheseal tenderness count (0–33)	2 (0–11)	1 (0–5)
Pain VAS (0–100 mm)^a^	65 (50–85)	35 (10–70)
Patient-reported symptomatic joint count (0–63)	12 (6–16)	5 (2–13)
Morning stiffness (min)	30 (0–60)	30 (0–90)

Continuous variables presented as median (interquartile range). Frequency presented as *n* (%).

a Scale from low to high severity. CHAQ: Childhood Health Assessment Questionnaire; VAS: visual analogue score.

None of the participants in the control group had active peripheral joints on clinical examination. One control participant fulfilled the criteria for clinical sacroiliitis based on SIJ tenderness and a positive Patrick’s test, albeit without inflammatory back pain.

There were 25/47 (53%) JIA patients with clinically detected joint inflammation (active JIA group); 23/47 participants with JIA had ACJ ≥ 1 and two participants had clinical sacroiliitis without other active joints. There were 22/47 (47%) patients with JIA without active joints or clinical sacroiliitis on examination (inactive JIA group). The characteristics, treatments and clinical disease activity measures of both JIA groups are detailed in Table 2.

**Table 2.** Disease characteristics, treatments, clinical and laboratory assessments in clinically active and inactive JIA groups

Variable	Inactive (*n* = 22)	Active (*n* = 25)
Disease duration, years	12 (6–15)	10 (6–14)
Enthesitis-related arthritis, *n* (%)	6 (27)	7 (28)
Extended oligoarticular, *n* (%)	5 (23)	7 (28)
Polyarticular RF negative, *n* (%)	4 (18)	5 (20)
Polyarticular RF positive, *n* (%)	2 (9)	2 (8)
Psoriatic, *n* (%)	2 (9)	2 (8)
Systemic, *n* (%)	2 (9)	1 (4)
Persistent oligoarticular, *n* (%)	1 (5)	1 (4)
Treatment with bDMARD and csDMARD	7 (32)	10 (40)
bDMARD monotherapy	7 (32)	3 (12)
csDMARD (one or more), *n* (%)	6 (27)	6 (24)
Anti-TNF-α	11 (50)	12 (48)
Tocilizumab	2 (9)	0 0
Rituximab	0 0	1 (4)
Canakinumab	1 (5)	0 0
MTX	10 (45)	12 (48)
SSZ	3 (14)	5 (20)
LEF	0 0	2 (8)
Steroids*, n* (%)	1 (5)	1 (4)
No DMARD, *n* (%)	2 (9)	6 (24)
NSAIDs (at least weekly)	4 (18)	16 (64)
Active joint count (0–79)	0 0	3 (2–4)
Limited joint count	0 (0–2)	4 (2–6)
PhGA (0–10)^a^	0 0	3 (3–5)
JADAS10-CRP^b^	2.0 (0.0–6.0)	13.0 (9.0–14.5)
PtGA (0–100 mm)^a^	20 (0–60)	52 (10–70)
CHAQ (0–3), *n* = 46	0.13 (0.00–1.00)	0.63 (0.00–1.44)
Entheseal tenderness count (0–33)	0 (0–4)	1 (0–5)
Pain VAS (0–100 mm)^a^	12.5 (0.0–55.0)	45.0 (20.0–75.0)
Patient-reported symptomatic joint count (0–63)	2.5 (0.0–10.0)	8.0 (3.0–14.0)
Morning stiffness (min)	2.5 (0.0–60.0)	60.0 (10.0–120.0)
ESR (mm/h), *n* = 45	2.0 (2.0–6.0)	6.0 (2.0–9.5)
CRP (mg/L)	0.6 (0.6–1.8)	1.5 (0.6–3.8)

Active: JIA participants with one or more active joint on examination or clinical sacroiliitis; inactive: JIA participants without active joints and clinical sacroiliitis.

a Scale from low to high severity.

b JADAS10-CRP was equal to JADAS71-CRP in all patients as AJC ≤ 10. AJC: active joint count bDMARD: biologic DMARD; CHAQ: Childhood Health Assessment Questionnaire; csDMARD: conventional synthetic DMARD; JADAS10: Juvenile Arthritis Disease Activity Score (maximum 10 active joints); LJC: limited joint count; PhGA: physician’s global assessment of disease activity; PtGA: patient’s global assessment of well-being; VAS: visual analogue score.

### Clinically and WBMRI detected joint inflammation: patient-level analysis

The WBMRI scan was scored positive for joint inflammation (one or more inflamed joint) in 28/47 (60%) patients with JIA and in 2/13 (15%) controls (Table 3).

**Table 3.** WBMRI-detected joint inflammation in JIA *vs* controls, and in active *vs* inactive JIA

JIA participants *vs* controls			
	JIA	Controls	Difference, % (95% CI)
WBMRI+	28 (60)	2 (15)	44 (20, 68)
WBMRI–	19 (40)	11 (85)	
Total	47 (100)	13 (100)	

Active *vs* inactive JIA participants			
	Active JIA	Inactive JIA	Difference, % (95% CI)
WBMRI+	19 (76)	9 (41)	35 (9, 62)
WBMRI–	6 (24)	13 (59)	
Total	25 (100)	22 (100)	

Data are presented as *n* (%). Active JIA: JIA participants with one or more active joint on examination or clinical sacroiliitis; inactive JIA: JIA participants without active joints and clinical sacroiliitis. Unpaired proportions were used for comparisons. WBMRI+: participants with inflammation in one or more joint by WBMRI; WBMRI–: participants without joint inflammation on WBMRI; WBMRI: whole-body MRI.

Of the patients with clinically active JIA, the WBMRI scan was positive in 19/25 (76%) patients. There were 6/25 (24%) patients with clinically active disease where the WBMRI scan was negative. Of the patients with clinically inactive JIA, the WBMRI scan was positive in 9/22 (41%). Eleven (50%) of the clinically inactive JIA patients fulfilled the Wallace criteria of clinically inactive disease; four of these patients had a positive WBMRI scan. The proportion of patients with positive WBMRI scan was higher in the clinically active JIA group than in the clinically inactive (Table 3).

The median [interquartile range (IQR)] number of joints with WBMRI-detected inflammation/patient was 1 (0–4) in the JIA group *vs* 0 (0) in the controls (*P* = 0.002). The median (IQR) number of joints with WBMRI-detected inflammation/patient was 3 (1–6) in the active JIA group *vs* 0 (0–1) in the inactive JIA group (*P* = 0.005).

### WBMRI-detected joint inflammation in clinically inactive joints: patient-level analysis

Subclinical joint inflammation was detected on WBMRI in one or more joint in 23/47 (49%) YP with JIA. It was detected in 14/25 (56%) patients with active JIA and in 9/22 (41%) patients with inactive JIA, (difference: 15%, 95% CI –13%, 43%).

The median (IQR) number of joints with subclinical inflammation/patient was 1 (0–4) in the active JIA group and 0 (0–1) in the inactive JIA group (*P* = 0.182).

### Relation between joint inflammation on WBMRI and disease activity measures in patients with JIA

The AJC, LJC, PhGA and JADAS10-CRP measurements were significantly higher in the JIA group with joint inflammation on WBMRI compared with the group without inflammation (Table 4). In comparison, we did not detect a statistically significant difference in the PtGA, CHAQ, CRP and ESR between the two groups. There was a positive correlation between the number of joints with WBMRI inflammation and AJC (rho = 0.52, *P* < 0.001), LJC (rho = 0.59, *P* < 0.001), PhGA (rho = 0.55, *P* < 0.001) and JADAS10-CRP (rho = 0.45, *P* = 0.002), (Supplementary Fig. S1, available at *Rheumatology* online).

**Table 4.** Characteristics and disease activity measures of JIA patients with and without joint inflammation on WBMRI

Variables	No joint inflammation on WBMRI (*n* = 19)	Joint inflammation on WBMRI (*n* = 28)	*P*-value
Patients’ demographics and disease characteristics			
Age, years	18.0 (17.0–20.0)	18.5 (16.0–20.5)	0.996
Male sex, *n* (%)	6 (32)	12 (43)	0.435
Disease duration, years	8 (6–13)	12 (6–15)	0.283
Biologic treatment, *n* (%)	12 (63)	15 (54)	0.514
Disease activity measures			
AJC (0–79)	0 (0–1)	2 (0–4)	**0.006**
LJC (0–75)	0 (0–2)	3 (1–6)	**0.001**
PhGA (0–10)	0.0 (0.0–2.0)	3.0 (0.5–4.0)	**0.002**
JADAS10-CRP	4.0 (0.7–8.3)	9.5 (4.0–14.2)	**0.041**
PtGA (0–100 mm)	25.0 (5.0–65.0)	28.5 (6.0–65.0)	0.953
CHAQ (0–3), *n* = 46	0.63 (0.00–1.13)	0.13 (0.00–1.50)	0.697
ESR (mm/h), *n* = 45	6 (2–9)	6 (2–8)	0.883
CRP (mg/l)	0.9 (0.6–1.8)	1.4 (0.6–3.6)	0.254
Other patient-reported outcomes			
Patient-reported symptomatic joint count (0–63)	9.0 (0.0–21.0)	4.5 (2.0–9.5)	0.718

Continuous variables presented as median (interquartile range). Comparison of continuous variables with Mann–Whitney *U* test and between categorical variables with Chi-squared test. *P*-values <0.05 marked in bold to indicate statistical significance. AJC: active joint count; CHAQ: Childhood Health Assessment Questionnaire; JADAS: Juvenile Arthritis Disease Activity Score; LJC: limited joint count; PhGA: physician’s global assessment of disease activity; PtGA: patient’s global assessment of well-being; WBMRI: whole-body MRI.

### Relation between joint inflammation on WBMRI and JIA subtype

The proportion of patients with WBMRI and clinically detected joint inflammation varied between the JIA subtypes (Supplementary Fig. S2, available at *Rheumatology* online).

All patients with polyarticular RF-negative JIA (*n* = 9) had joint inflammation on WBMRI, including at least one joint with subclinical inflammation. Subclinical inflammation was also found in 3/13 of ERA, 1/4 of polyarticular RF-positive, 1/4 of PsA, 1/3 of systemic, 1/2 of persistent and 7/12 of extended oligoarticular JIA patients.

### Clinically and WBMRI detected joint inflammation: joint-level analysis

**Figure 1.**
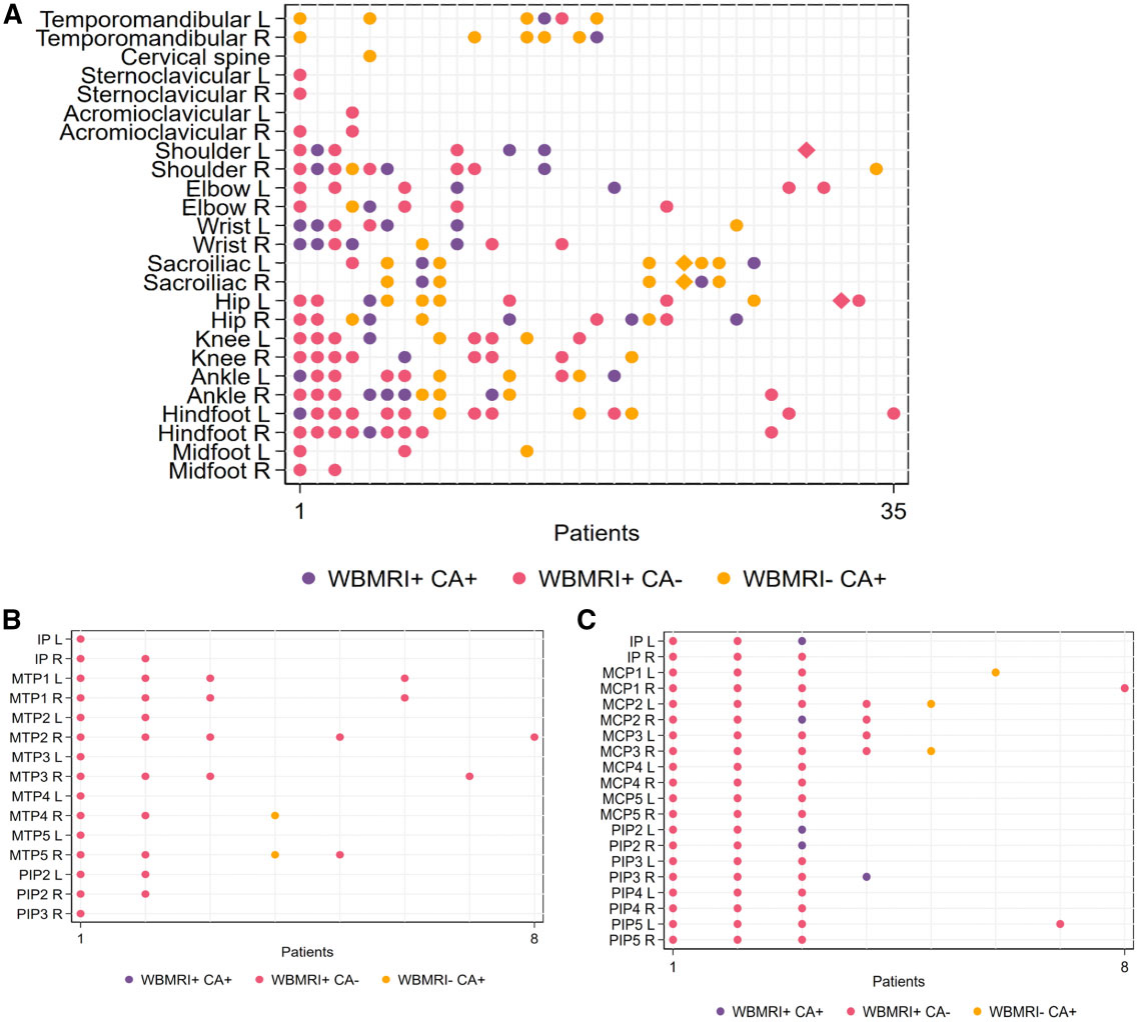
Inflammation detected by WBMRI and clinical assessment in 81 joints of JIA patients and controls. (**A**) In 25 joints/patient; (**B**) in hand joints (28 joints/patient); (**C**) in forefoot joints (28 joints/patient). Forty-seven patients with JIA and 13 controls were assessed. The joints assessed are shown on the *y*-axis and patients on the *x*-axis. Only patients/joints with joint inflammation by WBMRI or CA are shown on the *x*-axis and *y*-axis, respectively. Circles: patients with JIA; diamonds: controls. WBMRI+: joint inflammation on WBMRI; WBMRI–: no joint inflammation on WBMRI; CA+: joint inflammation on CA; CA–: no joint inflammation on CA; L: left; R: right; WBMRI: whole-body MRI; CA: clinical assessment

**Figure 2.**
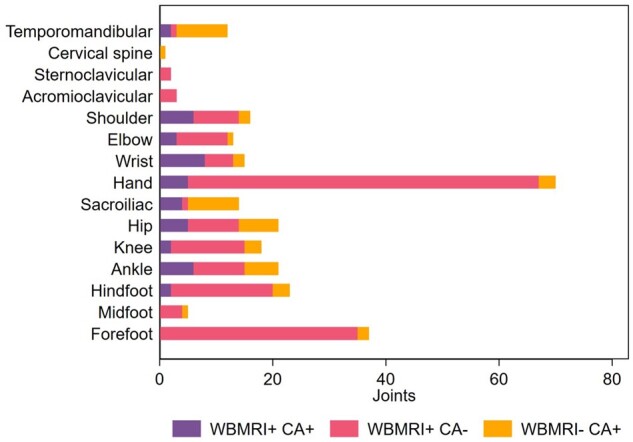
Agreement between CA and WBMRI examination per joint in patients with JIA. Patients with JIA were assessed for joint inflammation in 81 joints by WBMRI and CA. The findings of both methods per joint are shown as the number of joints in 47 patients. The hand and forefoot included the assessment of 28 small joints per patient respectively. WBMRI+: grade 2 synovitis (or other criteria for sacroiliitis and c-spine inflammation) on WBMRI; WBMRI–: no grade 2 synovitis (or no sacroiliitis or c-spine inflammation, respectively) on WBMRI; CA+: active joint on examination (or clinical sacroiliitis for sacroiliac joint); CA–: joint not active (or no clinical sacroiliitis for sacroiliac joint); CA: clinical assessment, WBMRI: whole-body MRI

The frequency of the WBMRI demonstrated inflamed joints compared with CA is shown for each joint at the patient level in Fig. 1. The total number of joints with inflammation by clinical *vs* WBMRI examination in all patients with JIA at the joint level is shown in Fig. 2. There were more hand and foot joints with discordant WBMRI and CA findings compared with other joints, however there was a higher joint count (*n* = 28) assessed in these regions per patient.

In the control group, joint inflammation was detected on WBMRI in one patient at the hip joint (1/13, 8%) and in the other patient at the shoulder joint (1/13, 8%).

### Peripheral enthesitis

Enthesitis in at least one peripheral (non-spinal) entheseal site was detected on WBMRI in 11/47 patients with JIA (23.4%) and in 1/13 (7.7%) control patients (difference: 15.7%, 95% CI –3.2%, 34.6%). There were more sites with enthesitis detected by clinical examination than WBMRI in both the JIA (*P* = 0.007) and control groups (*P* = 0.004). A detailed analysis on enthesitis is available in Supplementary Data S4, available at *Rheumatology* online.

### Structural damage

Structural damage in at least one joint was detected on WBMRI in 23/47 (49%) patients with JIA and in 1/13 (8%) controls (difference: 41%, 95% CI 21%, 62%).

The structural damage in the control group was detected at one joint (sacroiliac joint).

In descending frequency, structural damage was detected in patients with JIA on WBMRI in the SIJ (11/46, 24%), shoulder (7/47, 15%), wrist (5/47, 11%), ankle (4/47, 9%), elbow (3/44, 7%), TMJ (3/46, 7%), hip (2/46, 4%), knee (1/47, 2%) and hand joints (1/47, 2%).

### Relation between serum pro-inflammatory markers VEGF and MMP-3 and disease activity

Serum samples were collected from all JIA patients and 12/13 controls. One sample from the JIA and one from the control group were visibly haemolysed and excluded from the analysis. The concentrations of 7/12 analytes were within the standard detection range in ≥75% of participants (Supplementary Table S1, available at *Rheumatology* online).

The concentrations of MMP-3 and VEGF were higher in active than in inactive JIA patients (Supplementary Table S2, available at *Rheumatology* online). The MMP-3 and VEGF concentrations were greater in YP with JIA with WBMRI-detected joint inflammation than in those without, respectively (Supplementary Fig. S3, available at *Rheumatology* online). There was a correlation between MMP-3 and the number of joints with WBMRI-detected inflammation (rho = 0.50, *P* < 0.001) and between the latter and VEGF (rho = 0.32, *P* = 0.03) in patients with JIA. There was a correlation between MMP-3 and the clinical assessments AJC (rho = 0.47, *P* = 0.001) and JADAS10-CRP (rho = 0.43, *P* = 0.003), details in Supplementary Table S3, available at *Rheumatology* online.

## Discussion

In this study, we assessed YP with JIA and with non-inflammatory musculoskeletal pain (controls) for joint inflammation, enthesitis and structural damage using a structured WBMRI assessment as well as core JIA outcomes, routine laboratory markers and various serum biomarkers. We have shown that most patients with JIA (60%) had positive MRI scans for joint inflammation irrespective of their CA, and that there are very few positive WBMRI scans in the control group.

MRI-detected joint inflammation has been previously reported in children without inflammatory arthritis [19, 20]. The low frequency of joint inflammation in controls (in both control WBMRI+ YP only a single joint was inflamed) indicates that joint inflammation in most patients with JIA is likely related to their disease, rather than being a false-positive finding for reasons such as technical aspects of MRI [21] or misinterpretation of normal joint appearances in the immature skeleton [22]. In addition, we used a stringent definition of WBMRI-detected joint inflammation based on a combination of characteristics tailored to various anatomic areas, whereas other studies in JIA considered the presence of osteitis [13], synovial enhancement [23] or effusion alone [14] as active arthritis.

There were six JIA patients judged as clinically active where there was no inflammation seen on the MRI scan. This could be due to several factors including technical issues, such as joints not shown well on this form of imaging (e.g. TMJ). However, it is possible that the joints were not inflamed and there were other contributing factors to a positive CA, such as chronic pain, structural damage or factors related to the assessor.

In the JIA patients, we found a high frequency of clinically negative joints that were WBMRI positive. The significance of this finding is unknown, but it is possible that it represented true subclinical inflammation, especially as joint inflammation was associated with a biomarker signature in our study. Only a few retrospective studies have previously reported the frequency of subclinical synovitis detected by WBMRI in patients with JIA [12]. To our knowledge, this is the first study in JIA designed to minimize bias by including controls and blinding the readers to the patient’s diagnosis and clinical information. Our study detected subclinical joint inflammation in patients with clinically active and clinically inactive JIA. Further research is needed to explore the clinical significance of WBMRI-detected subclinical inflammation and its potential impact on JIA patient management adding to the information provided by clinical examination. The choice of treatment in JIA is influenced by the number [24] and type of joints affected [25, 26]. Therefore, identifying inflammation in additional or functionally and prognostically important joints on WBMRI may alter treatment plans in active patients. Moreover, we discovered that subclinical joint inflammation was present across all JIA subtypes but was remarkably detected in all patients with the polyarticular RF-negative JIA subtype. Polyarticular subtypes are associated with more persistently active disease than systemic or oligoarticular subtypes [27] and worse prognosis [28, 29].

We have shown that WBMRI-detected joint inflammation was associated with higher JADAS10-CRP and physician-reported core JIA outcomes (AJC, LJC and PhGA) in patients with JIA. This also suggests that WBMRI-detected inflammation is clinically relevant and supports the validity of WBMRI as a test to evaluate joint disease activity in JIA. Furthermore, the presence of joint inflammation on WBMRI and clinical examination was associated with higher serum concentrations of MMP-3 and VEGF, respectively, supporting a relation between imaging findings and the pathophysiological processes involved in JIA. Previous studies have demonstrated the role of MMP-3 and VEGF as biomarkers in JIA. Changes in MMP-3 correlated with changes in the swollen joint count in patients with ERA, a relation that was not detected for ESR in the same study [30]. Serum VEGF has been reported to correlate with the AJC in patients with JIA [31, 32] and be higher in patients with active disease compared with remission [33].

We did not detect an association between CHAQ and PtGA, or the inflammatory markers CRP and ESR, and joint inflammation on clinical examination or WBMRI. These patient-reported outcomes are essential for assessing disease activity and shared treatment decision-making in JIA. However, it is recognized that factors other than active inflammation can influence these outcomes, whereas WBMRI is an objective test for inflammation. A divergence between improving disease activity based on AJC and persistent symptoms according to patient-reported outcomes has previously been reported in a subset of JIA patients in UK [34] and Canadian [35] inception cohort studies. ESR was reported to be normal in most patients with active JIA in another study [36].

In our study, we did not detect a significant difference in the frequency of enthesitis in YP with JIA compared with controls by CA or WBMRI. The spatial resolution of WBMRI might be a limiting factor for detecting enthesitis. Additionally, the CA of enthesitis has low accuracy [37], which makes the evaluation of WBMRI’s performance difficult.

The control group in our study reported higher pain scores, and poorer function (measured by CHAQ) than patients with JIA. This was observed in patients with juvenile FM compared with paediatric patients with other rheumatic diseases in a large registry [38].

There are limitations in this study. There was a lack of previous literature data to enable us to calculate the study sample size. However, we detected statistically significant differences in the prevalence of joint inflammation in the JIA group *vs* controls, thus we believe these differences are very likely to be representative for larger populations. We recruited YP from one hospital, and we did not include patients younger than 14 years old. Therefore, the persistent oligoarticular and systemic JIA subtypes, which can be self-limiting in childhood, were underrepresented. The clinical and WBMRI assessment, as well as the imaging interpretation may be more challenging in younger children. We defined patients as ‘clinically inactive’ based on clinical examination and one-half of these patients fulfilled the Wallace criteria for inactive disease. These criteria are not validated for patients with psoriatic JIA and ERA, and we opted to use criteria that are applicable to all JIA subtypes.

This study did not investigate the prognostic significance of WBMRI-detected subclinical synovitis. A retrospective study showed that bone marrow oedema on MRI was associated with an increased risk of developing structural damage [19]. Further studies are needed to explore whether WBMRI-detected inflammation can predict flares and/or if a management pathway incorporating WBMRI in the assessment of patients can improve long-term patient outcomes compared with standard clinical practice.

Overall, the proportion of joints that could not be assessed on WBMRI was small (5%). However, this predominantly affected the elbow and forefoot joints which were not included in the field of view because of patients’ size.

This study has several strengths. Our WBMRI protocol was based on post-contrast images as the current state of non-contrast MRI techniques, such as diffusion-weighted imaging or short-tau inversion recovery (STIR), are not as sensitive for the detection of inflammation [39, 40] and less suitable for the assessment of small joints [41]. There is no validated scoring system for WBMRI in JIA, hence we developed a methodology to assess the musculoskeletal manifestations of JIA on WBMRI. We have evaluated the inter- and intra-reader agreement of our methodology and reported it separately. Recently, the OMERACT in JIA group proposed a scoring system for the WBMRI assessment of patients with JIA, based on non-contrast WBMRI scans [42]. Despite the differences between the two grading systems, the OMERACT group included the same joint pathologies as we did, with the caveat that non-contrast MRI scans cannot differentiate between synovial hypertrophy and joint effusion. We recognize that there is a need to develop non-contrast MRI techniques which will be less invasive and safer for patients, given the rare adverse effects of gadolinium.

### Conclusion

In conclusion, this study has demonstrated that WBMRI shows joint inflammation in around 60% of patients with JIA, and this is significantly greater than in controls, which had not been investigated before. This suggests that contrast WBMRI is a valid tool for the assessment of joint inflammation in YP with JIA. We found that subclinical joint inflammation was frequently detected in JIA patients with clinically active and inactive disease. WBMRI-detected joint inflammation was associated with clinical measures of disease activity and serum biomarkers. Further studies are required to evaluate the potential benefits of the use of WBMRI to support the clinical management of patients with JIA.

## Supplementary Material

keae039_Supplementary_Data

## Data Availability

The data underlying this article cannot be shared publicly due to ethical considerations of protecting the privacy of individuals who participated in the study. The data will be shared on reasonable request to the corresponding author.
