# Supplementary material for: Detection of inflammation by whole-body MRI in young people with juvenile idiopathic arthritis
Source: Rheumatology (Oxford). 2024 Jan 20;63(SI2):SI207–14. doi: 10.1093/rheumatology/keae039 (PMC11381681; doi:10.1093/rheumatology/keae039)
Supplement: keae039_Supplementary_Data [file keae039_supplementary_data.docx]

**SUPPLEMENTARY MATERIAL**

**Title: Detection of inflammation by whole-body MRI in young people with juvenile idiopathic arthritis (JIA)**

**Authors: Varvara Choida^1,2,3^, Timothy J.P. Bray^1,4^, Niels van Vucht^4^, Maaz Ali Abbasi^4^, Alan Bainbridge^1,5^, Thomas Parry^1^, Debajit Sen^2,3^, Sue Mallett^1^, Coziana Ciurtin^2,3^, Margaret A. Hall-Craggs^1,4^**

**Affiliations**
1. Centre for Medical Imaging, University College London, University College London, London, UK.

2. Centre for Adolescent Rheumatology, Division of Medicine, University College London, Rayne Building, London WC1E 6JF, UK

3. Department of Rheumatology, University College London Hospitals NHS Foundation Trust, London, London, UK

4. Department of Imaging, University College London Hospitals NHS Foundation Trust, London, UK

5. Department of Medical Physics, University College Hospitals Trust, London, UK

Correspondence to: Professor Margaret Hall-Craggs, Centre for Medical Imaging, University College London, 2nd Floor Charles Bell House, 43-45 Foley Street, W1W 7TS London, UK. Email: m.hall-craggs@ucl.ac.uk

**Supplementary Data S1**

**Entheseal tenderness assessed in 33 sites per patient**

Plantar fascial insertion at calcaneus, plantar fascial insertion at the base of the fifth metatarsal joint, Achilles tendon insertion at calcaneus, quadriceps femoris insertion at the 2 o’clock superior portion of patella, quadriceps femoris insertion at the 10 o’clock superior portion of patella, patellar ligament insertion at inferior pole of patella or tibial tuberosity, hip extensor insertion at greater trochanter, anterior superior iliac spine, Iliac crest, ischial tuberosity, posterior superior iliac spine, 5th lumbar spinous process, medial and lateral humeral epicondyle, supraspinatus insertion, 1^st^ and 7^th^ costochondral joints.

**Supplementary Data S2**

**Scanning parameters of whole-body MRI protocol**

Imaging acquisition was divided in 6-8 anatomical stations, including an optional station for imaging the feet in tall patients. The images from the first seven stations were combined into a whole-body image for each of the four sets of Dixon images (water-only, fat-only, in-phase and opposed-phase).

The scanning settings were TE: 1.31-1.32 ms, TR: 3.5 ms, flip angle: 10 degrees, acquisition matrix: 68-172 x 235-320 x 120 (depending on the station), voxel size 1.59-1.6 x 1.59-1.75 x 5 mm^3^, and interslice gap: -2.5.

**Imaging review**

The post-contrast water-only Dixon images were assessed for the presence of joint inflammation, structural damage, enthesitis and spinal inflammation. The in-phase images were used to evaluate the presence of structural damage in peripheral joints (in addition to fat-only images for sacroiliac joints).

Grade 1 (G1) synovitis was defined as the presence of above-normal intensity post-contrast synovial enhancement without any of the above characteristics and grade 0 (G0) as normal intensity post-contrast synovial enhancement. G0 and G1 synovitis were considered negative for joint inflammation.

The cervical spine (c-spine) was assessed for inflammation and structural damage as one joint, to mirror the clinical assessment in patients with JIA.

C-spine was assessed for anterior and posterior inflammation and for synovitis in the atlantoaxial (AA) and atlantooccipital (AO) joints. Anterior spinal inflammation was defined as post-contrast enhancement at the vertebral corners or adjacent to the vertebral endplates. Posterior spinal inflammation was defined as facet joint inflammation, or enthesitis of spinal ligaments. If any of the above was positive at any level, then the joint was considered positive for joint inflammation.

Acute sacroiliitis was defined as subchondral or periarticular bone marrow oedema.

Enthesitis was defined as the presence of above-normal enhancement within the bone marrow (osteitis), tendon or ligament, at the site of the entheses’ insertion.

Structural damage in peripheral joints was assessed dichotomously and defined as the presence of erosions or joint remodelling in peripheral joints.

Structural damage at the c-spine was defined as erosions, fat infiltration, ankylosis or new bone formation, malalignment of AO, AA or subaxial joints.

Chronic sacroiliitis was defined as the presence of fat infiltration/metaplasia, erosions, ankylosis, or subchondral sclerosis.

**Assessable joints on WBMRI**

The non-assessable joints included 13% of the small forefoot joints and 9% of elbows as they were not included in the field of view. The proportion of assessable joints on the other joints was ≥ 95%. In total, 237/4860 (4.9%) joints could not be assessed by all readers.

**Supplementary Data S3**

**Serum analysis**

The serum was extracted after centrifuging the clotted blood samples at 1,200 gravitation force for 10 minutes and stored at -80 °C. The samples were thawed once at the Platform Immune Monitoring MultiPlex Core Facility (PIM-MPCF) within the University Medical Centre Utrecht (UMCU) where the serum concentrations were measured by a multiplex immunoassay based on Luminex xMAP technology (x: analyte, MAP: Multi-Analyte Profiling).


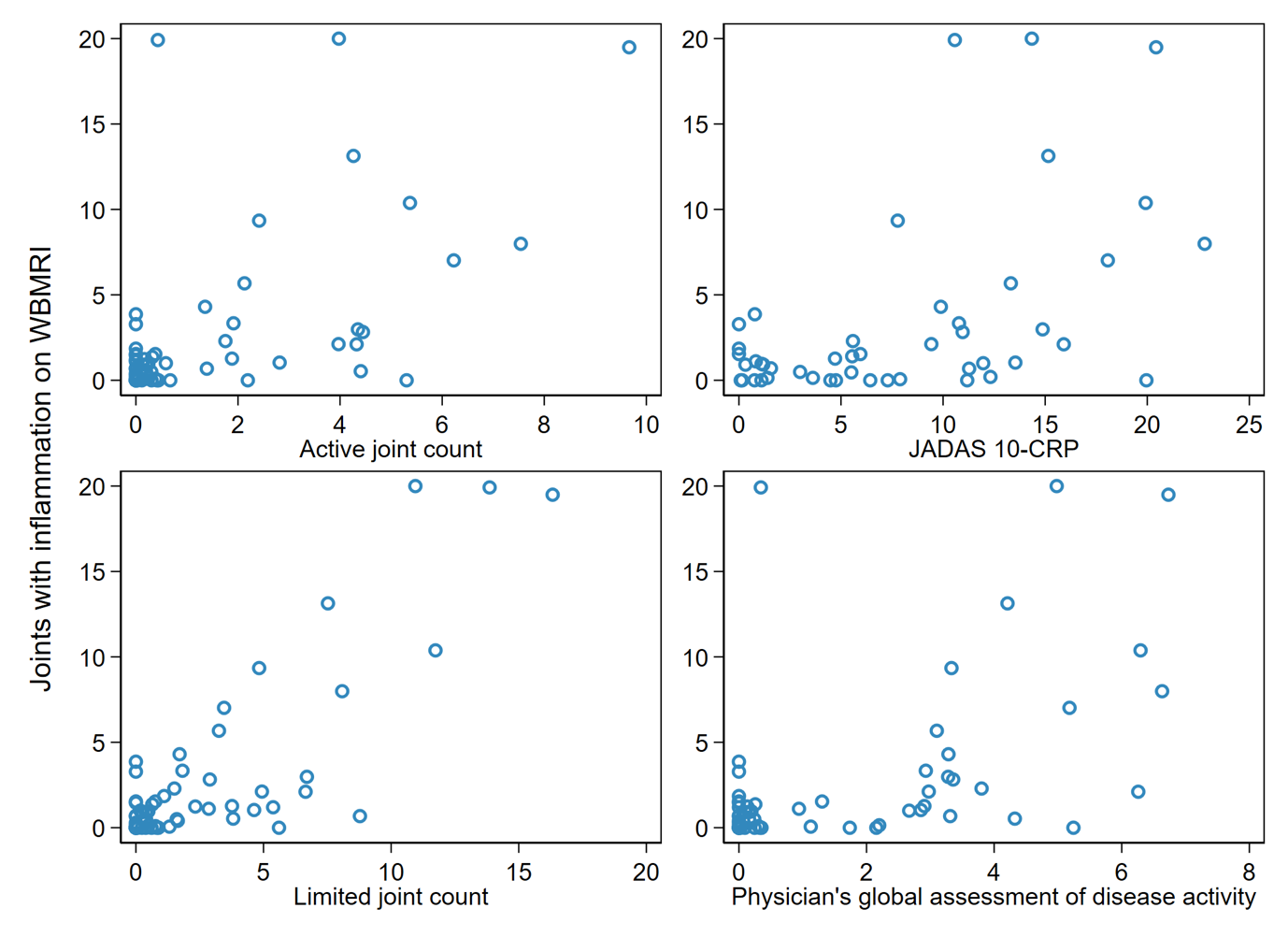


**Supplementary Figure S1.** Scatter plots of joint inflammation on WBMRI and clinical disease activity measures in JIA patients. Scatter plots of the number of joints with inflammation/patient detected on WBMRI (y-axis) and disease activity measures (x-axis) in 47 patients with JIA (blue circles). Three patients with WBMRI inflammation joint count above 20 (36, 43, 54) are depicted as having 20 inflamed joints to better visualise the spread of values for the majority of patients. The physician’s global assessment of disease activity (0-10) increases with disease activity. JADAS 10-CRP: Juvenile Arthritis Disease Activity Score based on maximum of 10 active joints and CRP, JIA: juvenile idiopathic arthritis, WBMRI: whole-body MRI.

**
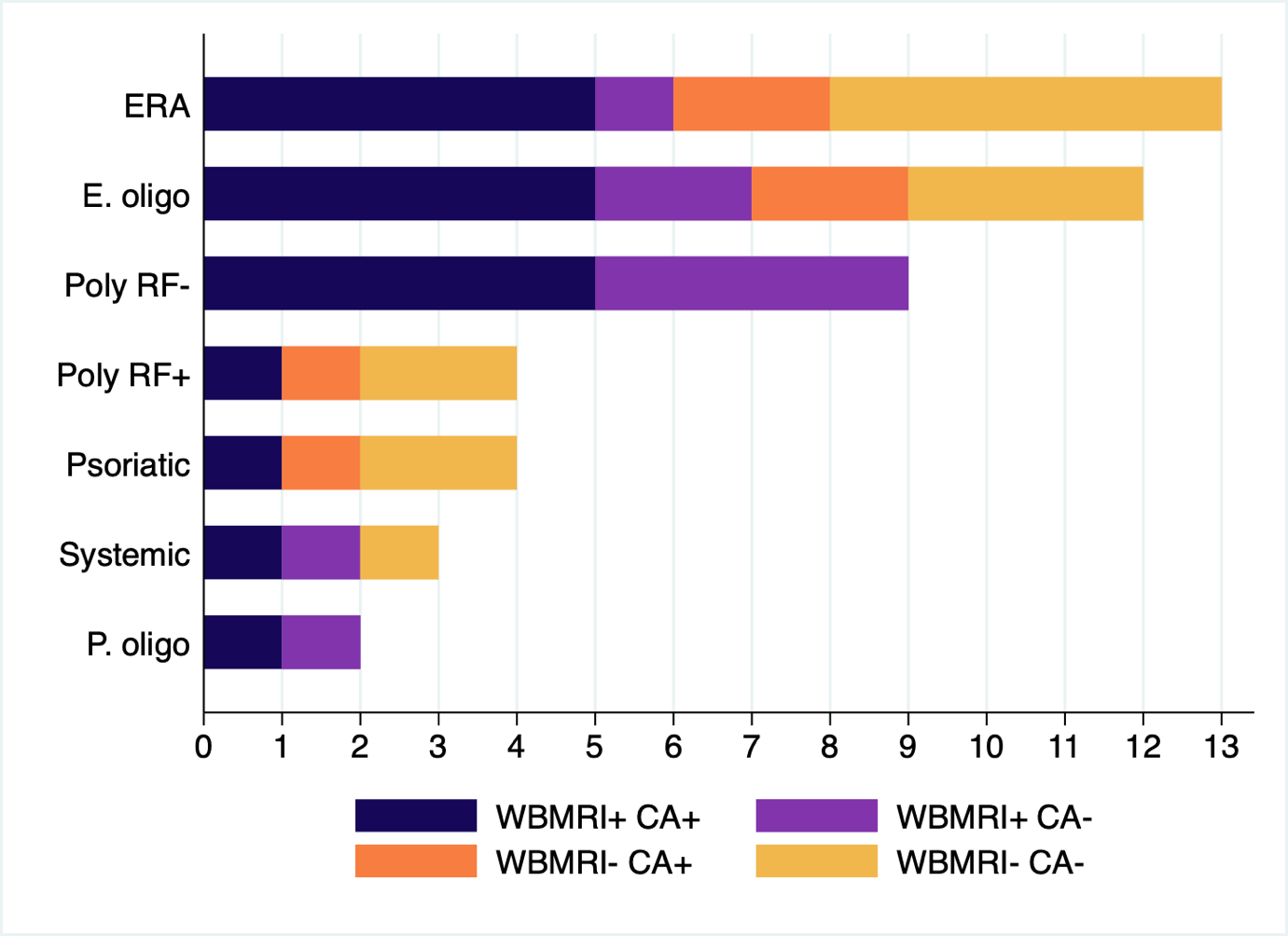
**

**Supplementary Figure S2.** Frequency of detection of joint inflammation by WBMRI vs CA across JIA subtypes. Patients with JIA (N=47) were assessed for the presence or absence of joint inflammation by WBMRI and CA. The frequency of findings is presented per JIA subtype. WBMRI+: ≥1 joint with inflammation on WBMRI, WBMRI-: no joint with inflammation on WBMRI, CA+: ≥1 joint with inflammation on CA, CA-: no joint with inflammation on CA, CA: clinical assessment, E.oligo: extended oligoarticular, ERA: enthesitis-related arthritis, JIA: juvenile idiopathic arthritis, P. oligo: persistent oligoarticular, Poly RF-: polyarticular rheumatoid factor negative, Poly RF+: polyarticular rheumatoid factor positive, WBMRI: whole-body MRI

**Supplementary Data S4**

**Enthesitis**

Clinical examination

Entheseal tenderness in at least one site per participant was found in 26/47 (55%) young people (YP) with JIA and in 9/13 (69%) controls (difference: -14%, 95% CI -43%, 15%). Entheseal tenderness was found in 16/25 (64%) patients in the clinically active group and in 10/22 (45%) patients in the clinically inactive JIA group (difference:19%, 95% CI -10%, 47%).

Enthesitis on WBMRI in active vs inactive JIA patients

Enthesitis was detected in 7/25 (28%) patients in the active JIA group and 4/22 (18%) patients in the inactive JIA group (difference: 10%, 95% CI -14%, 34%).

Comparison of enthesitis detected by clinical assessment and by WBMRI in YP with JIA

The number of sites with enthesitis per patient by clinical examination was significantly higher than the number of sites with enthesitis per patient by WBMRI (P=0.007, Wilcoxon matched-pairs signed-rank test).

- More specifically, more sites with enthesitis were detected by WBMRI than clinical examination in 6/47 YP (13%) with JIA; 5 of those 6 YP with JIA had no sites with enthesitis on clinical examination.
- The same number of sites with enthesitis per patient were detected by both methods in 19/47 (40%) YP with JIA; 16 of those 19 YP had no sites with enthesitis by both methods.
- Fewer sites with enthesitis per patient were detected by WBMRI than clinical examination in 22/47 (47%) YP with JIA; 20 of those 22 YP with JIA had no sites with enthesitis on WBMRI.

Comparison of enthesitis detected by clinical assessment and by WBMRI in controls

The median (interquartile range) of the sites with enthesitis per participant was 2 (0-11) on clinical examination and 0 (0) on WBMRI in controls. The difference in the number of sites with enthesitis per patient with both methods was statistically significant (P=0.004, Wilcoxon matched-pairs signed-rank test).

- More specifically, none of the controls had a higher number of sites with enthesitis on WBMRI than clinical examination.
- Four controls (31%) had no sites with enthesitis by both methods
- Nine controls (69%) had fewer sites with enthesitis on WBMRI than clinical examination; eight of those nine controls had no sites with enthesitis detected on WBMRI.

Sites with enthesitis on WBMRI

The sites with enthesitis in JIA patients by WBMRI were in descending frequency the greater trochanter (11 sites in 6 patients), ischial tuberosity (8 sites in 5 patients), elbow olecranon (3 sites in 2 patients), calcaneus (2 sites with plantar fasciitis in 2 patients), and anterior inferior iliac spine (2 sites in one patient).

The only site with enthesitis detected on WBMRI in one control patient was the greater trochanter.

Frequency of enthesitis per JIA subtype

Enthesitis by clinical examination was detected in all JIA subtypes. More specifically, the frequency was 6/13 in ERA, 9/14 in oligoarticular (only in extended oligoarticular), 5/9 in polyarticular RF-negative, 2/4 in polyarticular RF-positive, 2/4 in psoriatic and 2/3 in systemic JIA subtype.

Peripheral enthesitis on WBMRI was noted in 4 patients with extended oligoarticular, 4 patients with polyarticular RF-negative, 2 patients with ERA one patient with psoriatic JIA subtypes.

**Conclusions**

1. We did not detect a statistically significant difference in the frequency of enthesitis by WBMRI between young people with JIA and controls, or between active and inactive JIA patients. Enthesitis was detected rarely on WBMRI in all patient groups, therefore a larger patient number might be needed to detect differences between the patient groups.
2. There were more sites with enthesitis detected by clinical examination than WBMRI in both the JIA and control groups. The same finding has been reported by most studies assessing patients with inflammatory arthritis with WBMRI and clinical examination(1).
3. Enthesitis was not identified more frequently in patients with ERA or psoriatic JIA than in other JIA subtypes. In contrast, a previous inception cohort study reported the presence of clinical enthesitis across all the non-systemic JIA subtypes but with a higher frequency in patients with ERA(2).
4. There was no significant difference between the frequency of clinical enthesitis, defined as entheseal tenderness in at least one of the 33 sites assessed per participant, between the YP with JIA and controls. This finding taken together with the significantly lower number of sites with enthesitis on WBMRI than clinical examination in the control group, suggests that the clinical assessment of enthesitis lacks specificity. Other stricter definitions have been suggested for the diagnosis of clinical enthesitis in JIA, which either involve a medical decision that the enthesitis requires treatment(3), or the involvement of multiple sites and/or the persistence of signs over time(2).

**Supplementary Table S1*.*** Frequency of measured concentrations within the standard range or beyond (extrapolated or undetectable) per analyte.

| **Samples with/without detectable levels** | **IL-6** | **IL-17** | **IL-23** | **IL-33** | **TNF -alpha** | **IFN -gamma** | **MIF** | **G-MCSF** | **S100A8** |
| --- | --- | --- | --- | --- | --- | --- | --- | --- | --- |
| Below range of detection | 2  (3.5) | 32 (56.1) | 7 (12.3) | 35 (61.4) | 31 (54.4) | 16 (28.1) | 6 (10.5) | 0 | 0 |
| Extrapolated concentration | 6 (10.5) | 6 (10.5) | 7 (12.3) | 2 (3.5) | 2  (3.5) | 3  (5.3) | 0 | 53 (93) | 1  (1.8) |
| Within standard range | 49 (86.0) | 19 (33.3) | 43 (75.4) | 20 (35.1) | 24 (42.1) | 38 (66.7) | 51 (89.5) | 4  (7) | 56 (98.2) |
| C/I/A | 10/20/25 | 4/10/  11 | 8/20/  22 | 2/9/  11 | 4/8/  14 | 8/15/  18 | 9/19/  23 | 11/21/25 | 11/21/  25 |
| Results presented as n samples (%). Each sample belongs to a different patient. Concentrations measured by Luminex Xmap Technology. MMP-3, VEGF and CD40L concentrations were within standard range of detection in all samples. C/I/A: n samples with ‘extrapolated’ and ‘within standard range’ concentrations in the control /inactive JIA/ active JIA groups, CD40L: CD40 ligand, G-MCSF, granulocyte-macrophage colony-stimulating factor, IFN-gamma: interferon gamma, IL-6: interleukin 6, IL-17: interleukin 17, IL-23: interleukin 23, IL-33: interleukin 33, MIF: macrophage migratory inhibitory factor, MMP-3: MMP-3: matrix metalloproteinase-3, n: number, S100A8: S100 calcium-binding protein A8, TNF-alpha: tumour necrosis factor – alpha, VEGF: vascular endothelial growth factor. | | | | | | | | | |

**Supplementary Table S2.** Serum markers’ levels in controls and JIA participants (with clinically active and inactive disease)

| **Pro-inflammatory proteins** | **N** | **Inactive JIA** | **Active JIA** | **P-value** | **Controls** | **JIA** | **P-value** |
| --- | --- | --- | --- | --- | --- | --- | --- |
| IL-6  (pg/ml) | 55 | 32  (7.5-72.8) | 20  (6.6-72.4) | 0.659 | 11.8  (5.2-26.2) | 27.3  (6.6-72.4) | 0.168 |
| IL-23  (pg/ml) | 50 | 827.5 (157.8-2720.5) | 329.4 (32.9-2179.3) | 0.477 | 552.2 (121.9-2274.6) | 687.1 (67.2-2553.7) | 0.928 |
| S100A8  (pg/ml) | 57 | 4227.9 (3784.3-4839.5) | 5098.7 (4139.6-6623.0) | 0.133 | 5270.6 (3851.2-6036.0) | 4622.2 (3784.3- 5782.2) | 0.421 |
| MIF  (pg/ml) | 51 | 622.8 (441.4-1302.8) | 1037.7 (600.9-2735.8) | 0.076 | 810.8  (463.7 -961.4) | 867.8 (515.9-1613) | 0.484 |
| CD40L  (pg/ml) | 58 | 3170.8 (2492.3-4153.1) | 4274.4 (2311.4-4997.7) | 0.428 | 3426.3 (2257.9-5247.0) | 3823.6 (2334.4-4740.4) | 0.428 |
| VEGF  (pg/ml) | 58 | 127  (89.7-210.4) | 262.3 (153.8-371.1) | **0.039** | 146.6  (64-382.8) | 204.8 (94.2-308.8) | 0.944 |
| MMP-3  (ng/ml) | 58 | 19.6 (13.6- 29.5) | 38.3 (34.6- 64.3) | **<0.001** | 16.3 (8.3-26.1) | 31.6  (19.6- 44.4) | **0.001** |
| Medians (interquartile range). Concentrations measured by Luminex Xmap Technology. Samples from 21 inactive JIA, 25 active JIA and 11 controls, excluding samples with concentrations below level of detection (Supplementary Table 1). Extrapolated values were included. There were no excluded or extrapolated values for CD40L, VEGF and MMP-3. Analytes included if ≥75% samples within standard range. Active and inactive JIA as per clinical examination. Bold P-values indicate statistical significance (P<0.05), Wilcoxon rank-sum test. CD40L: CD40 ligand, IL-6: interleukin 6, IL-23: interleukin 23, MIF: macrophage migratory inhibitory factor, MMP-3: matrix metalloproteinase-3, S100A8: S100 calcium-binding protein A8, VEGF: vascular endothelial growth factor. | | | | | | | |


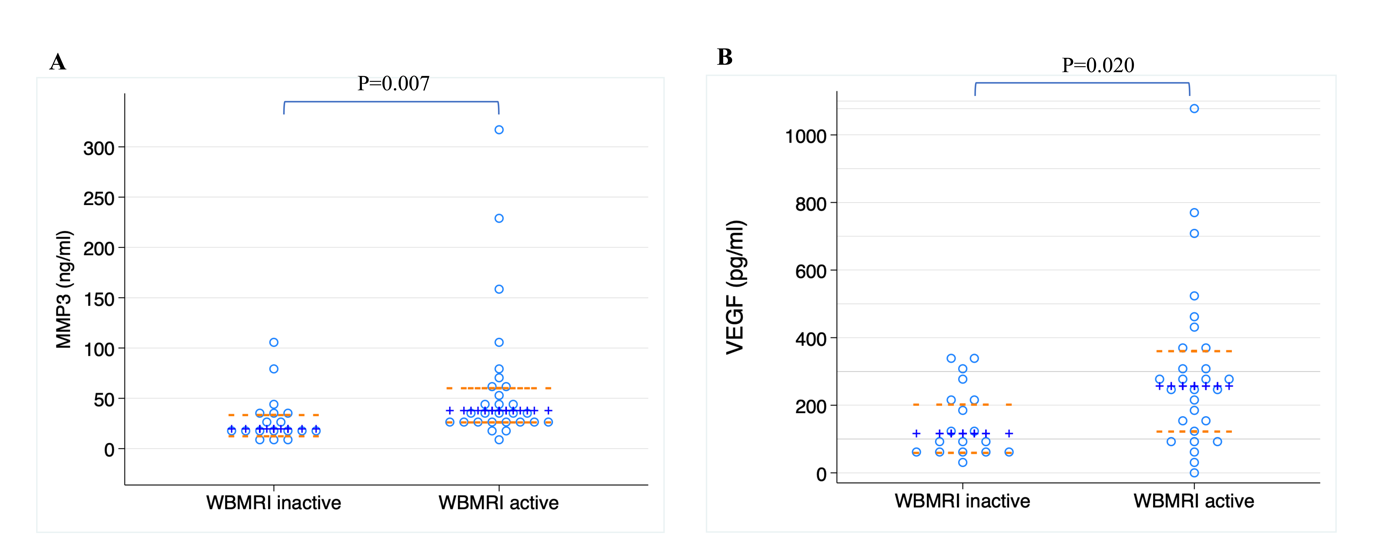


**Supplementary Figure S3.** Serum biomarkers in JIA patients with and without WBMRI-detected joint inflammation (n=46). A. MMP-3 concentrations were higher in WBMRI active vs WBMRI inactive group [median (IQR), ng/ml: 37.8 (28.8-62.6) vs 19.6 (15.0-36.1)]. B. VEGF concentrations were higher in WBMRI active vs WBMRI inactive group [median (IQR), pg/ml: 256.9 (130.6-367.6) vs 116.4 (67.0-210.4)]. WBMRI active: ≥ 1 inflamed joint on WBMRI, WBMRI inactive: no inflamed joints on WBMRI. Serum concentrations (hollow circles), medians (plus signs), IQR (dashed lines). P<0.05: statistical significance (Mann-Whitney U test). JIA: juvenile idiopathic arthritis, IQR: interquartile range, MMP-3: matrix metalloproteinase-3. VEGF: vascular endothelial growth factor, WBMRI: whole-body MRI.

**Supplementary Table S3.** Spearman's rank correlation between serum pro-inflammatory proteins MMP-3 and VEGF and measures of disease activity

| **Disease activity measures** | | **Spearman’s rank coefficient (rho), P-value** | | |
| --- | --- | --- | --- | --- |
| N=46 | **MMP-3** | | **VEGF** | |
| AJC (0-79) | | **0.47, P=0.001** | | 0.26, P=0.085 |
| JADAS10-CRP | | **0.43, P=0.003** | | 0.19, P=0.212 |
| WBMRI joint count with inflammation | | **0.50, P<0.001** | | **0.32, P=0.030** |
| CRP | | 0.19, P= 0.202 | | -0.05, P=0.740 |
| ESR, n=44 | | 0.10, P= 0.537 | | 0.14, P= 0.350 |
| P-value < 0.05 defined as statistically significant and presented in bold. WBMRI joint count with inflammation was the total number of joints with inflammation in 81 joints per patient with JIA on WBMRI. AJC: active joint count, CRP: c-reactive protein, ESR: erythrocyte sediment ratio, JADAS10-CRP: Juvenile Arthritis Disease Activity Score based on maximum of 10 active joints and CRP, MMP-3: matrix metalloproteinase-3, VEGF: vascular endothelial growth factor, WBMRI: whole-body MRI. | | | | |

**References**

1. Choida V, Madenidou AV, Sen D, Hall-Craggs MA, Ciurtin C. The role of whole-body MRI in musculoskeletal inflammation detection and treatment response evaluation in inflammatory arthritis across age: A systematic review. Semin Arthritis Rheum. 2022;52:151953.

2. Rumsey DG, Guzman J, Rosenberg AM, Huber AM, Scuccimarri R, Shiff NJ, et al. Characteristics and Course of Enthesitis in a Juvenile Idiopathic Arthritis Inception Cohort. Arthritis Care Res (Hoboken). 2018;70(2):303-8.

3. Ringold S, Angeles-Han ST, Beukelman T, Lovell D, Cuello CA, Becker ML, et al. 2019 American College of Rheumatology/Arthritis Foundation Guideline for the Treatment of Juvenile Idiopathic Arthritis: Therapeutic Approaches for Non-Systemic Polyarthritis, Sacroiliitis, and Enthesitis. Arthritis Rheumatol. 2019;71(6):846-63.
